# Supplementary material for: Geometric constraints on human brain function
Source: Nature. 2023 May 31;618(7965):566–74. doi: 10.1038/s41586-023-06098-1 (PMC10266981; doi:10.1038/s41586-023-06098-1)
Supplement: Supplementary file 1 — This file contains Supplementary Information, including Figs. 1–11, Tables 1–3 and additional references. [file 41586_2023_6098_MOESM1_ESM.docx]

Supplementary Information for

Geometric constraints on human brain function

James C. Pang^1^*^†^, Kevin M. Aquino^2†^, Marianne Oldehinkel^3^, Peter A. Robinson^2^, Ben D. Fulcher^2^, Michael Breakspear^4^, Alex Fornito^1^

^1^The Turner Institute for Brain and Mental Health, School of Psychological Sciences, and Monash Biomedical Imaging, Monash University, Australia

^2^School of Physics, University of Sydney, Australia

^3^Donders Institute for Brain, Cognition, and Behaviour, Radboud University Medical Centre, Netherlands

^4^School of Psychological Sciences, University of Newcastle, Australia

*Corresponding author. Email: [james.pang1@monash.edu](mailto:james.pang1@monash.edu)

^†^These authors contributed equally to this work

Table of Contents

[S1. Neural field theory 2](#_Toc131769763)

[S2. Human Connectome Project data 2](#_Toc131769764)

[S2.1. Task-evoked data 2](#_Toc131769765)

[S2.2. Task-free resting-state data 3](#_Toc131769766)

[S2.3. Connectome data 3](#_Toc131769767)

[S3. Individual-specific cortical eigenmodes 3](#_Toc131769768)

[S4. Effect of connectome thresholding on connectome eigenmodes 4](#_Toc131769769)

[S5. Comparison between geometric eigenmodes and a functionally derived basis set 5](#_Toc131769770)

[S6. Comparison between geometric eigenmodes and Fourier basis sets 6](#_Toc131769771)

[S7. Modal power spectra of surrogate maps 8](#_Toc131769772)

[S8. NFT wave model 9](#_Toc131769773)

[S9. Optimization of the BEI neural mass model 11](#_Toc131769774)

[S10. Lag threads algorithm 12](#_Toc131769775)

[S11. Supplementary Discussion 13](#_Toc131769776)

[S12. Supplementary References 13](#_Toc131769777)

[S13. Supplementary Figures 17](#_Toc131769778)

[S14. Supplementary Tables 24](#_Toc131769779)

[S15. Supplementary Video 25](#_Toc131769780)

S1. Neural field theory

Neural field theory (NFT) is a class of biophysical models that explain mesoscale to macroscale brain dynamics (from about 0.5 mm to whole brain) as the outcome of spatially extended, time-varying fields of neural activity ^1–7^. General NFTs focus on local average dynamics of neural populations, such as mean firing rates and soma voltages. Important biophysical processes, such as dendritic and synaptic processes, axonal conduction delays, and summation of dendritic current in the cell soma, are incorporated as local population averages through a series of principled mathematical reductions.

NFTs treat neural tissue at scales above ~0.5 mm as a spatial continuum with local properties and point-to-point white-matter connectivity that decreases smoothly with distance. Although more general cases can be treated via NFT, including interactions with subcortical structures ^7,8^, the simplest versions include only the cortex and assume that its connectivity is homogeneous and isotropic, with the connection strength only depending on the distance between points and typically decreasing approximately exponentially with their separation ^4,9–11^. This relation is especially relevant in the context of functional MRI (fMRI), where dynamics are relatively slow and the strength of active connections has a spatial dependence that is approximately exponential ^9,11,12^. The resulting spatiotemporal evolution of the activity fields has been effectively described by a physiologically constrained variant of NFT developed by Robinson and colleagues ^1,2,5,6,13^ as comprising damped waves propagating across the cortical sheet after being excited by external inputs and local cortical or corticothalamic dynamics. Over the last two decades, this NFT formulation has successfully explained and unified a diverse range of experimental phenomena in a unified way, including but not limited to electroencephalography (EEG) spectra ^14,15^, evoked potentials ^16,17^, arousal states ^18,19^, whole-brain effective connectivity ^12^, cortical activity waves ^20^, and sleep state reorganisations ^21^. Because mesoscale to macroscale brain dynamics are approximately operating in the linear regime under normal conditions (excluding seizure-like dynamics) ^22^, the Robinson et al. NFT has shown that spatial eigenmodes (modes) of activity naturally emerge in the brain ^23,24^, with their properties shaped by the brain’s intrinsic geometry and topology ^20,25^. These modes are the topic of interest in this study. We refer the readers to the extensive literature of NFTs for a more detailed discussion (see for example ^4–8,23,26–29^ and the references cited therein).

S2. Human Connectome Project data

S2.1. Task-evoked data

We analysed task-evoked fMRI data measured in 7 task domains that have been shown to reliably recruit a wide array of neural systems ^30^. The 7 tasks were: social, motor, gambling, working memory (wm), language, emotion, and relational. Supplementary Table 2 shows the specific contrasts involved in each task domain and the key contrast investigated in this study. In total, we analysed 47 contrasts, which include the 7 key contrasts. The key contrasts represent those used commonly in the literature to map the major activation pattern elicited by the task. See ^30^ for details about each task and contrast. The analysis was performed on individual task-activation maps computed via FSL’s (<https://fsl.fmrib.ox.ac.uk/>) cross-run (Level 2) FEAT analysis ^31^. We used the task maps as provided by HCP with minimal smoothing (2 mm) mapped onto the fsLR-32k CIFTI space, with 32,492 vertices in each hemisphere, via multimodal surface matching ^32^.

S2.2. Task-free resting-state data

We analysed task-free resting-state fMRI acquired in one scanning session using a left to right (LR) encoding direction. The scan lasted for 14.4 min with a total of 1200 time frames. In brief, the resting-state fMRI acquisition parameters were: isotropic voxel size of 2 mm, repetition time (TR) of 720 ms, and echo time (TE) of 33.1 ms. All other acquisition parameters can be found in ^33^. Each individual’s data were preprocessed by HCP via their minimal preprocessing pipeline ^34^ and were also subjected to ICA-FIX to correct for structured noise and residual confounds ^35^. No additional smoothing was performed. Similar to the task-evoked data, the resting-state data were mapped onto the fsLR-32k CIFTI space. Hence, each individual’s data were represented as a matrix of size 32,492 vertices $\times$ 1200 time frames on each hemisphere.

S2.3. Connectome data

To derive the connectome eigenmodes, we used individual connectomes derived from diffusion MRI (dMRI) data via probabilistic tractography, as provided in ^36^. In brief, the dMRI acquisition parameters were: isotropic voxel size of 1.25 mm, TR of 5520 ms, TE of 89.5 ms, b-weightings of 1000, 2000, 3000 s/mm^2^, and 6 b0-scans. All other acquisition parameters can be found in ^33^. Each individual’s data were preprocessed by HCP via their diffusion preprocessing pipeline (v3.19.0) ^34^. To generate the connectomes, tractograms were generated using MRtrix with probabilistic tractography, 5 million streamlines that connect anatomically distinct brain regions, multi-shell multi-tissue (MSMT) constrained spherical deconvolution (CSD) anatomically constrained tractography (ACT), and 2nd-order Integration over Fiber Orientation Distributions algorithm (iFOD2). See ^36^ for further details. The fsLR-32k cortical surface mesh in standard MNI space was used to define the grey-matter–white-matter interface. For each individual, streamlines generated in each hemisphere were mapped to the closest vertices on the surface mesh to construct a high-resolution weighted connectome (32,492 $\times$ 32,492 matrix size and weights representing the total number of streamlines). See ^36^ for further details.

S3. Individual-specific cortical eigenmodes

The Helmholtz equation,

$$\begin{aligned} \nabla^{2}\psi=\Delta\psi=-\lambda\psi,\#\left( S1 \right) \end{aligned}$$

can be used to solve the eigenmodes of any cortical surface mesh model, but changes in the geometry of the mesh can alter the resulting eigenvalues, $\lambda$, and eigenmodes, $\psi$. Hence, the eigenvalues and eigenmodes derived from individual subject surfaces, particularly at very short wavelengths, will differ and cannot be straightforwardly compared ^37,38^. For simplicity, here we used the eigenmodes generated from a common template surface (see ‘Derivation of cortical geometric eigenmodes’ in Methods). While this allows for comparison of data reconstructions in different individuals, it obscures any potential effects associated with individual differences in cortical geometry. However, Supplementary Fig. 3 shows that using geometric eigenmodes derived from individual cortical surfaces does not change the general results of the study, suggesting that, for present purposes, the eigenmodes derived from the population-averaged template surface represent a good approximation of fundamental geometric eigenmodes. Nonetheless, to highlight certain nuances of individual geometry, we show in Supplementary Fig. 4 that 200 individual-specific eigenmodes perform slightly better than template-derived eigenmodes in some individuals, especially in reconstructing task-activation maps but not in reconstructing resting-state activity. However, Supplementary Fig. 5 shows that the results for individual-specific and template-derived eigenmodes eventually converge at very short wavelengths (~500^th^ mode). Thus, the effects of individual differences in cortical geometry on brain function are captured by the first 200 modes, which is consistent with recent work ^38^ and corresponds to a very small fraction of the maximum possible number of eigenmodes (~0.6%).

S4. Effect of connectome thresholding on connectome eigenmodes

Many network properties depend on network connection densities, and our specific thresholding procedure to generate the group-averaged connectome, $A_{C}$, is somewhat arbitrary. Hence, we also derived connectome eigenmodes for variants of $A_{C}$ mapped across a range of thresholds, from 1% to 13% (i.e., the density of the unthresholded connectome) in increments of 2%. The results in Supplementary Fig. 6 show that connection density did indeed affect the reconstruction accuracy of connectome eigenmodes, such that sparser matrices were associated with improved reconstructions. Given that long-range connections tend to have lower weights and are thus more likely to be thresholded away first, the application of more conservative thresholds emphasizes local, exponential distance rule (EDR)-like connectivity until finally converging on the surface mesh. These results thus align with the view that reconstruction performance is generally improved when focusing mostly on local connectivity. Notably, finely sampling connection densities from 0.01% to 5% reveals a putatively optimal threshold between 0.4% and 0.7% that results in peak reconstruction accuracy for 200 connectome eigenmodes (Supplementary Fig. 7). This optimal threshold may represent a physiologically plausible trade-off between sensitivity and specificity in tract reconstruction with dMRI. However, the reconstruction accuracy never surpasses the accuracy of the geometric eigenmodes. The sensitivity of the reconstruction accuracies obtained with connectome eigenmodes to the density threshold further underscores the complexity of this approach in comparison to geometric eigenmodes, which require no choices regarding a specific threshold.

Our focus on high-resolution, vertex-level connectomes is intended to allow fair comparison with previous work examining the efficacy of connectome eigenmodes in reconstructing functional data ^39,40^. However, many other studies on connectomes first apply a discrete parcellation to the data and investigate the properties of connectomes generally mapped at the level of ~10^2^ to ~10^3^ parcellated brain regions ^41,42^. For completeness, we also derived eigenmodes using connectomes parcellated at different resolutions, generated using the Schaefer400, Schaefer600, Scahefer800, and Schaefer1000 parcellations. These parcellations were chosen because they have at least 200 parcels per hemisphere, allowing us to calculate at least 200 connectome eigenmodes for direct comparison with the 200 geometric eigenmodes used in the study. Note, however, that this approach ignores the spatial embedding of the brain, resulting in inferior reconstruction accuracy when compared to geometric eigenmodes (Supplementary Fig. 8). This result again confirms the importance of capturing local connectivity and regional geometry when deriving an appropriate anatomical basis set for brain function.

S5. Comparison between geometric eigenmodes and a functionally derived basis set

To further evaluate the efficacy of geometric eigenmodes in representing brain activity, we compared their performance to a basis set derived via a principal component analysis (PCA) of functional data itself. Since PCA defines a linearly optimal decomposition of the functional data, we sought to evaluate the out-of-sample generalizability of this approach by applying the PCA to a training set of 200 individuals and validating its reconstruction accuracy on a held-out test set of 55 individuals. The number of individuals for the training set was chosen to produce 200 principal components (PCs), matching the number of geometric eigenmodes analysed in the study. Note that the PCs were arranged in order of the variance in the data that they explain.

For the task-evoked data, we constructed 7 training datasets, one for each of the 7 key task contrasts in Supplementary Table 2. Each training dataset was constructed by forming a matrix of size 32,492 vertices $\times$ 200 individuals with each task’s activation data. We then applied a spatial PCA to obtain 200 spatial PCs, describing common modes of variance in spatial activation patterns across individuals (Supplementary Fig. 9).

For the task-free resting-state data, a similar approach can be implemented, where a training dataset is constructed by temporally concatenating the data of individuals in the training set, producing a matrix of size 32,492 $\times$ 240,000 (i.e., 240,000 = 1200 time frames $\times$ 200 individuals). However, performing a standard PCA on this very large data matrix carries a high computational burden. We therefore used the MELODIC Incremental Group-PCA (MIGP) method, which has been shown to produce outputs that closely approximate those of a standard PCA in a fully-concatenated dataset using a much more computationally efficient method ^43^. In summary, MIGP temporally concatenates the data of a small number of individuals (here we used two individuals) to construct the matrix $W$. It then uses $W$ to construct a time frame $\times$ time frame covariance matrix and applies an eigenvalue decomposition to extract the top $m$ temporal eigenvectors (here we used $m$ = 1200), and multiplies the eigenvectors with $W$ to obtain $m$ weighted spatial eigenvectors, yielding a new $W$. Then, $W$ is incrementally updated by concatenating it with the next individual’s data and the entire process above is repeated to obtain the new $W$ representing $m$ spatial eigenvectors. This iterative method was performed until all the individuals in the training set were considered. We retained the top 200 spatial PCs from $W$.

The PCs obtained from the training sets in the task-evoked and task-free resting-state data were then used to decompose the data of the individuals in the test set and calculate the corresponding out-of-sample reconstruction accuracy. Note that the performance of the geometric eigenmodes is inherently out-of-sample, since the modes were derived from a cortical surface mesh representation that is independent of the fMRI datasets considered here.

We emphasize that components derived from PCA only capture statistical properties of the data (i.e., maximal variance) and do not provide any mechanistic insights; i.e., they are statistical or phenomenological descriptions of the data and are ordered according to variance explained without regard for spatial wavelength. They are thus fundamentally different from geometric eigenmodes, which are directly linked to the structural properties of the system, are informed by a generative model of how brain structure gives rise to function ^23^, and are arranged according to their spatial frequencies or wavelengths. As shown in Extended Data Fig. 4, this distinction leads to an early performance advantage for PCA over geometric eigenmodes, in which the first few PCs can more accurately capture the data than the first few geometric eigenmodes. This is because the PCs do not have the same ordering constraint as the geometric eigenmodes; i.e., early PCs are free to span any spatial wavelength, as dictated by the optimal decomposition of the data, whereas geometric eigenmodes are ordered from long to short wavelengths, by construction. However, Extended Data Fig. 4 also shows that there is a limit on the accuracy with which the PCs can reconstruct out-of-sample data, likely because higher-order PCs capture idiosyncratic properties of the training data. In contrast, adding more geometric eigenmodes improves the model’s capacity to resolve reproducible shorter-wavelength features of both the training and test data, such that the out-of-sample reconstruction surpasses the accuracy of the PCs across most comparisons by about 40–50 modes.

S6. Comparison between geometric eigenmodes and Fourier basis sets

To further confirm that the performance of geometric eigenmodes is not trivially driven by how the mathematics of any basis set expansion, we compared the geometric eigenmodes to six simple spatial Fourier basis sets based on combinations of sines and/or cosines. The Fourier basis sets were constructed using the following real-valued functions:

$$\begin{aligned} F_{1}:= \cos\left( \frac{2\pi\left( j_{x}-1 \right)x}{L_{x}}+\frac{2\pi\left( j_{y}-1 \right)y}{L_{y}}+\frac{2\pi\left( j_{z}-1 \right)z}{L_{z}} \right),\#\left( S2 \right) \end{aligned}$$

$$\begin{aligned} F_{2}:= C_{1}\cos\left( \frac{2\pi(j_{x}-1)x}{L_{x}} \right)+C_{2}\cos\left( \frac{2\pi(j_{y}-1)y}{L_{y}} \right)+C_{3}\cos\left( \frac{2\pi(j_{z}-1)z}{L_{z}} \right),\#\left( S3 \right) \end{aligned}$$

$$\begin{aligned} F_{3}:= C_{1}\cos\left( \frac{2\pi\left( j_{x}-1 \right)x}{L_{x}}+\frac{2\pi\left( j_{y}-1 \right)y}{L_{y}}+\frac{2\pi\left( j_{z}-1 \right)z}{L_{z}} \right)+ \\ C_{2}\sin\left( \frac{2\pi\left( j_{x}-1 \right)x}{L_{x}}+\frac{2\pi\left( j_{y}-1 \right)y}{L_{y}}+\frac{2\pi\left( j_{z}-1 \right)z}{L_{z}} \right),\#\left( S4 \right) \end{aligned}$$

where $j_{x}$, $j_{y}$, $j_{z}$ are integer constants, $x$, $y$, $z$ are the spatial positions of each point on the spherical surface mesh representation of the cortex, $L_{x}$, $L_{y}$, $L_{z}$ are the periods in each direction, and $C_{1}$, $C_{2}$, $C_{3}$ are fitting constants. For each function, we constructed a regular and an irregular version, resulting in six basis sets. The regular version corresponds to when $j_{x}=j_{y}=j_{z}=j$ such that the spatial wavelengths of mode $j$ in the x-, y-, and z-directions are regularly spaced and increase by $\frac{2\pi}{L_{x}}$, $\frac{2\pi}{L_{y}}$, $\frac{2\pi}{L_{z}}$ as the mode number increases, which is a standard implementation in Fourier analysis. The irregular version corresponds to when $j_{x}$, $j_{y}$, $j_{z}$ are integer combinations and not necessarily equal; hence, the spatial wavelengths in the x-, y-, and z-directions are irregularly spaced. We implemented this version because it affords the Fourier basis sets greater freedom and thus the best possible chance of performing well. To associate a ($j_{x}, j_{y}, j_{z})$ combination to a single mode $j$, we arranged the combinations in order of increasing $j_{x}+j_{y}+j_{z}$. For example, the ($j_{x}, j_{y}, j_{z})$ combination for the first ten modes follows the set $\left\{ \left( 1,1,1 \right), \left( 1,1,2 \right),\left( 1,2,1 \right),\left( 2,1,1 \right),\left( 1,2,2 \right),\left( 2,1,2 \right),\left( 2,2,1 \right),\left( 1,1,3 \right),\left( 1,3,1 \right),\left( 3,1,1 \right) \right\}$. These two versions allow us to explore how the spatial wavelengths can affect the decompositions of the Fourier basis sets, given that the spatial wavelengths of the geometric eigenmodes are irregularly spaced (see Supplementary Table 1).

Next, we defined the periods to be $L_{i}:=[max \left( i \right)-\min\left( i \right)]$ for $i=x$,$y$,$z$, to have a heuristic that respects the shape of the brain. In addition, the $-1$ in ($j_{i}-1)$ensures that the first mode is constant to make it comparable to the first geometric mode. The forms in Eqs. (S3) and (S4) have more degrees of freedom, with the fitting constants, $C_{1}$, $C_{2}$, $C_{3}$, estimated separately, allowing us to weight the cosine and/or sine functions differently. Therefore, mode $j$ now has multiple amplitudes to be estimated during the mode decomposition instead of just one. These added degrees of freedom result in an increased model complexity relative to geometric eigenmodes; more specifically, the Fourier basis sets formed by Eqs. (S3) and (S4) involve estimating 2 and 3 coefficients per mode, respectively, whereas geometric eigenmodes require fitting just one coefficient per mode.

Although other forms and complicated choices can be made to construct a Fourier basis set, the above choices are well motivated by their simplicity, yielding unique, real-valued spatial modes, and cover the key implementation choices one could make. Extended Data Fig. 5a shows the spatial profiles of the resulting modes with unit coefficients, which we used to reconstruct task-activation maps and resting-state data (similar to Fig. 1d). Extended Data Fig. 5b shows that geometric eigenmodes significantly outperform the Fourier basis sets in reconstructing both task-evoked and resting-state data, further emphasizing that the accurate representation provided by geometric eigenmodes is not a trivial result of any basis set expansion. Moreover, this conclusion remains regardless of how the spatial wavelengths of the modes in the x-, y-, and z-directions are defined. To reiterate, even though the Fourier basis sets based on Eqs. (S3) and (S4) have more degrees of freedom to fit the data when compared to geometric eigenmodes, geometric eigenmodes still show superior performance, underscoring their parsimony in accounting for brain dynamics.

We present this analysis of Fourier basis sets for completeness and to demonstrate that such functions cannot accommodate the boundary conditions of non-regularly shaped objects, such as the cortical surface (e.g., Dirichlet or Neumann conditions for the medial wall) ^44^. This is because Fourier basis sets can only be properly constructed for objects with regular shapes (e.g., rectangle) and are more suitable for analysing functions defined on such shapes (e.g., 2D images). Hence, for the cortical surface defined on a Riemannian manifold, using a Fourier basis set is generally not well-motivated and using the eigenmodes of the LBO is more appropriate ^45,46^. In fact, LBO eigenmode analysis on the Riemannian manifold is formally considered a generalization of Fourier analysis ^47^. The same applies to discrete networks, such as a connectome; for this reason, the eigendecomposition of the graph Laplacian (as done in the work) is commonly used in the field of spectral graph theory as an alternative to a classical Fourier transform ^48^. Thus, whilst it is possible to decompose spatial maps of brain activity using Fourier basis sets, they are highly inefficient and poorly suited to the current problem. We therefore do not advocate their use. We also note that Fourier basis sets offer no insights into the generative processes underlying brain activity. Our primary focus is to compare physiologically principled and anatomically constrained basis sets (i.e., geometric and connectome eigenmodes) to uncover the critical constraints on brain dynamics, and not to identify statistically optimal basis sets.

S7. Modal power spectra of surrogate maps

Typical image processing pipelines induce some degree of spatial smoothing, which serves to filter out high-frequency (short-wavelength) spatial patterns of activity. To ensure that the dominant long-wavelength power we identified in our analyses was not merely the product of spatial smoothing induced by fMRI preprocessing, we analysed the power spectra of surrogate random data with varying levels of smoothing. We first generated 10,000 random maps in volume space taken from a zero-mean Gaussian distribution. We then smoothed the surrogate maps at kernel sizes with full-width at half-maximum (FWHM) ranging from 0 to 50 mm. Finally, we projected the surrogate maps onto the fsLR-32k CIFTI space (via Nilearn) and analysed their modal power spectra. Note that we performed the smoothing in volumetric instead of surface space to mimic prevailing neuroimaging processing practices, in which data processing is commonly applied to volumetric data before results are resampled to the cortical surface ^49^. This procedure thus also incorporates any smoothing induced by the resampling procedure.

We compared the modal power spectra of the empirical activation maps and the surrogate maps by calculating the mean square logarithmic error (MSLE),

$$\begin{aligned} \mathrm{MSLE}=\sqrt{\frac{1}{N}\sum_{j=1}^{N=200} \left[ \log_{10} \left( P_{j}^{\mathrm{empirical}} \right)-\log_{10} \left( P_{j}^{\mathrm{surrogate}} \right) \right]^{2}},\#\left( S5 \right) \end{aligned}$$

where $P_{j}^{\mathrm{empirical}}$ and $P_{j}^{\mathrm{surrogate}}$ is the power in mode $j$ in the empirical and surrogate maps, respectively. The MSLE is more effective as a distance measure than the typical mean square error because the power of long- to short-wavelength modes have scales that vary across several orders of magnitude.

The coloured lines in Extended Data Fig. 8a show the modal power spectra of the surrogate data, Extended Data Fig. 8b shows the average distance (i.e., MSLE) between empirical and surrogate spectra averaged across contrasts, and Extended Data Fig. 8c shows MSLE separately obtained for each task contrast (instead of averaged across contrasts). We found that smoothing kernels smaller than 10 mm do not adequately capture the concentration of spectral power in long spatial wavelengths of empirical maps (Fig. 3a and Extended Data Fig. 8a). In fact, the surrogate data needed an average kernel size of 20 mm (best fits obtained from the minimums in Extended Data Fig. 8b) to achieve a spectrum comparable to the empirical data, which is considerably larger than the size of the kernels used in neuroimaging processing (~8 mm in volume space ^49^ and ~15 mm in surface space ^50^). Similar results were obtained when task contrasts were separately analysed instead of their average, with minimum MSLE showing minor variations around the mean (solid lines in Extended Data Fig. 8c). These findings thus confirm that the long-wavelength content of empirical activation maps cannot be explained by preprocessing alone. Moreover, whilst spatially extended patterns of task activations can also potentially be recovered using simple smoothing functions and avoiding statistical thresholding, the geometric eigenmode approach provides much deeper understanding of the mechanisms underlying the activations because the modes can be directly linked to rigorous biophysical models of brain dynamics as established by NFT (see for example ^6,7,14,23^). They also offer a natural multiscale characterization of the data.

S8. NFT wave model

The simple NFT wave model described by an isotropic damped wave equation without regeneration is ^5,6,51^,

$$\begin{aligned} \left[ \frac{1}{\gamma_{s}^{2}}\frac{\partial^{2}}{\partial t^{2}} + \frac{2}{\gamma_{s}}\frac{\partial}{\partial t}+1-r_{s}^{2}\nabla^{2} \right]\phi\left( \boldsymbol{r},t \right)=Q(\boldsymbol{r},t),\#\left( S6 \right) \end{aligned}$$

where $\phi(\boldsymbol{r},t)$ is the neural activity at location $\boldsymbol{r}$ and time $t$, $Q$ is an external input, $\gamma_{s}$ is the damping rate, and $r_{s}$ is the spatial length scale of the wave propagation. The model assumes that the propagation of activity between points is governed by their white-matter connectivity, with strength that decays approximately exponentially with distance. This distance-dependence is more apparent when Eq. (S6) is converted into its equivalent integral form as follows. Consider two points on the neocortex at locations $\boldsymbol{r'}$ and $\boldsymbol{r}$ connected by a white-matter tract (see visual schematic in Supplementary Fig. 10). The activity $\phi(\boldsymbol{r},t)$ at location $\boldsymbol{r}$ and time $t$ can be considered as a spatiotemporal convolution of the source $Q(\boldsymbol{r'},t')$ at location $\boldsymbol{r'}$ and time $t'$ and the white-matter-based connectivity kernel $W\left( \boldsymbol{r},t;\boldsymbol{r}^{\boldsymbol{'}},t^{'} \right)$; i.e.,

$$\begin{aligned} \phi\left( \boldsymbol{r},t \right)=\int W\left( \boldsymbol{r},t;\boldsymbol{r}^{\boldsymbol{'}},t^{'} \right)Q\left( \boldsymbol{r}^{\boldsymbol{'}},t^{'} \right)d^{2}\boldsymbol{r}^{\boldsymbol{'}}dt^{'}.\#\left( S7 \right) \end{aligned}$$

In the isotropic case, $W$ depends only on the spatial separation between points and the time difference such that $W\left( \boldsymbol{r},t;\boldsymbol{r}^{\boldsymbol{'}},t^{'} \right):=W\left( \boldsymbol{r}-\boldsymbol{r}^{\boldsymbol{'}},t-t^{'} \right)$. The kernel $W$ is also known as the Green’s function, which is the activity $\phi$ due to a point source. Using the Green’s function method ^52^, previous work has shown that an appropriate expression for $W$, such that it becomes a solution of the damped wave equation in Eq. (S6) with an intense point source $Q\left( \boldsymbol{r},t;\boldsymbol{r}^{\boldsymbol{'}},t^{'} \right)=\delta(\boldsymbol{r}-\boldsymbol{r}^{\boldsymbol{'}})\delta(t-t^{'})$ and for $\left| \boldsymbol{r}-\boldsymbol{r}^{'} \right|\leq\gamma_{s}r_{s}(t-t^{'})$, is ^4–6,13^

$$\begin{aligned} W\left( \boldsymbol{r}-\boldsymbol{r}^{\boldsymbol{'}},t-t^{'} \right)=\frac{\gamma_{s}}{r_{s}}\frac{\exp\left( \frac{-|\boldsymbol{r}-\boldsymbol{r}^{'}|}{r_{s}} \right)}{\sqrt{\gamma_{s}^{2}r_{s}^{2}\left( t-t^{'} \right)^{2}-\left| \boldsymbol{r}-\boldsymbol{r}^{'} \right|^{2}}}\Theta\left[ \gamma_{s}r_{s}\left( t-t^{'} \right)-\left| \boldsymbol{r}-\boldsymbol{r}^{'} \right| \right],\#\left( S8 \right) \end{aligned}$$

where $|\boldsymbol{r}-\boldsymbol{r}^{\boldsymbol{'}}|$ is the white-matter tract distance between points, $\Theta$ is the Heaviside step function, and $\gamma_{s}$ and $r_{s}$ are the damping rate and spatial length scale, respectively, as defined in Eq. (S6). See ^6^ for a detailed derivation of Eq. (S8). Equation (S8) thus demonstrates how the wave dynamics defined by Eq. (S6) can be directly related to an underlying isotropic anatomical connectivity that decays exponentially with distance. In particular, solving the damped wave equation in differential form in Eq. (S6) is equivalent to solving the integral equation,

$$\begin{aligned} \phi\left( \boldsymbol{r},t \right)=\frac{\gamma_{s}}{r_{s}}\int\frac{\exp\left( \frac{-|\boldsymbol{r}-\boldsymbol{r}^{'}|}{r_{s}} \right)}{\sqrt{\gamma_{s}^{2}r_{s}^{2}\left( t-t^{'} \right)^{2}-\left| \boldsymbol{r}-\boldsymbol{r}^{'} \right|^{2}}}\Theta\left[ \gamma_{s}r_{s}\left( t-t^{'} \right)-\left| \boldsymbol{r}-\boldsymbol{r}^{'} \right| \right]Q\left( \boldsymbol{r}^{\boldsymbol{'}},t^{'} \right)d^{2}\boldsymbol{r}^{\boldsymbol{'}}dt^{'},\#\left( S9 \right) \end{aligned}$$

with the latter explicitly showing the spatiotemporal effect invoked by white-matter connectivity of a characteristic range of $r_{s}$ (~84 mm ^6^). There are other more complex variations of the wave equation in Eq. (S6), but this simple version of the model evaluates the basic physical process that could account for empirical data. Incorporation of spatial heterogeneities or structured input into the wave equation in Eq. (S6) is a topic for future investigation.

Despite the simplicity of the model, the full spatiotemporal solution of Eq. (S6) at 32,492 vertices is computationally expensive. We therefore used the following approach to efficiently solve Eq. (S6) on the cortex. First, we assumed that $\phi$ is time-space separable because the structure of the system is constant in time, such that

$$\begin{aligned} \phi\left( \boldsymbol{r},t \right)=\phi(t)\phi\left( \boldsymbol{r} \right).\#\left( S10 \right) \end{aligned}$$

By substituting Eq. (S10) into Eq. (S6), the term $\nabla^{2}\phi\left( \boldsymbol{r},t \right)$ becomes $\phi(t)\nabla^{2}\phi\left( \boldsymbol{r} \right)$. Note that $\nabla^{2}\phi\left( \boldsymbol{r} \right)$ is the same as the left-hand side of the Helmholtz equation in Eq. (S1); hence, the spatial component of the traveling wave solution, $\phi\left( \boldsymbol{r} \right)$, on the cortical surface can be written as a combination of geometric eigenmodes $\psi_{j}(\boldsymbol{r})$. Therefore, Eq. (S10) can be written as

$$\begin{aligned} \phi\left( \boldsymbol{r},t \right)=\sum_{j=1}^{N} \phi_{j}\left( t \right)\psi_{j}(\boldsymbol{r}),\#\left( S11 \right) \end{aligned}$$

where $\psi_{j}\left( \boldsymbol{r} \right)$ is mode $j$, $\phi_{j}\left( t \right)$ is the time-varying component of mode $j$, and $N$ is the number of modes. Second, we employed mode decomposition on the input $Q(\boldsymbol{r},t)$ such that

$$\begin{aligned} Q\left( \boldsymbol{r},t \right)=\sum_{j=1}^{N} q_{j}\left( t \right)\psi_{j}(\boldsymbol{r}),\#\left( S12 \right) \end{aligned}$$

where $q_{j}\left( t \right)$ is the time-varying amplitude obtained via mode decomposition (see ‘Mode decomposition of brain activity’ in Methods). Important consequences of the above separation into temporal and spatial factors are that the spatial modes are not sensitive to the local temporal dynamics, nor to the value of $r_{s}$ so long as it is small compared to the radius of curvature of the surface.

Substituting Eqs. (S11), (S12), and (S1) into Eq. (S6), we obtain

$$\begin{aligned} \sum_{j=1}^{N} \left[ \frac{1}{\gamma_{s}^{2}}\frac{\partial^{2}}{\partial t^{2}} + \frac{2}{\gamma_{s}}\frac{\partial}{\partial t}+1+r_{s}^{2}\lambda_{j} \right]\phi_{j}\left( t \right)\psi_{j}\left( \boldsymbol{r} \right)=\sum_{j=1}^{N} q_{j}\left( t \right)\psi_{j}(\boldsymbol{r}),\#\left( S13 \right) \end{aligned}$$

where $\lambda_{j}$ is the eigenvalue corresponding to mode $j$. Therefore, the ordinary differential equation for solving the time-varying component of mode $j$, $\phi_{j}\left( t \right)$, is

$$\begin{aligned} \left[ \frac{1}{\gamma_{s}^{2}}\frac{d^{2}}{dt^{2}} + \frac{2}{\gamma_{s}}\frac{d}{dt}+1+r_{s}^{2}\lambda_{j} \right]\phi_{j}\left( t \right)=q_{j}\left( t \right).\#\left( S14 \right) \end{aligned}$$

One can solve Eq. (S14) via numerical methods, but it can be solved more simply – and exactly – by taking its Fourier transform vs. time, thereby yielding the algebraic equation,

$$\begin{aligned} \left[ -\frac{\omega^{2}}{\gamma_{s}^{2}}-\frac{2i\omega}{\gamma_{s}}+1+r_{s}^{2}\lambda_{j} \right]\phi_{j}\left( \omega\right)=q_{j}\left( \omega\right),\#\left( S15 \right) \end{aligned}$$

where $\omega$ is the temporal angular frequency, $\phi_{j}(\omega)$ is the Fourier transform of $\phi_{j}(t)$, and $q_{j}\left( \omega\right)$ is the Fourier transform of $q_{j}(t)$. Therefore, the solution $\phi_{j}(t)$ has the form,

$$\begin{aligned} \phi_{j}\left( t \right)=\mathcal{F}^{-1}\left\{ \phi_{j}(\omega) \right\}=\mathcal{F}^{-1}\left\{ \frac{{\gamma_{s}^{2}q}_{j}\left( \omega\right)}{-\omega^{2}-2i\omega\gamma_{s}+\gamma_{s}^{2}\left( 1+r_{s}^{2}\lambda_{j} \right)} \right\},\#\left( S16 \right) \end{aligned}$$

where $\mathcal{F}^{-1}$ is the inverse Fourier transform operator. Finally, Eq. (S16) can be substituted into Eq. (S11) to obtain the neural activity $\phi\left( \boldsymbol{r},t \right)$ at every vertex.

As noted above, the spatial part of Eq. (S6) satisfies the Helmholtz equation in Eq. (S1). This differential equation is equivalent to an integral form (Eq. (S9)), where the activity at a given point gives rise to activity elsewhere with a weight that represents the connectivity that is implicit in NFT. In the cortical case, this corresponds to an exponential decrease with distance––i.e., an EDR-like spatial dependence––consistent with experimental evidence ^6^. Hence, the use of geometric eigenmodes implicitly incorporates an EDR-like connectivity, which also includes long-range connections, but does not directly account for topologically complex connections not conforming to a simple exponential rule.

S9. Optimization of the BEI neural mass model

The BEI neural mass model has 15 fixed parameters and 4 free parameters, i.e., $w_{EE}$, $w_{EI}$, $w_{IE}$, and $G$, that were optimized to fit the data, following ^53^. Briefly, feedback inhibition control ^54^ was implemented to adjust $w_{IE}$ to set the firing rate $r_{i}^{(E)}$ of all regions to approximately 3 Hz. Hence, the analytic expression for $w_{IE}$ at the steady-state conditions $\left\langle S^{\left( E \right)} \right\rangle\approx0.17$ nA and $\left\langle I^{\left( E \right)} \right\rangle\approx0.38$ nA is

$$\begin{aligned} w_{IE}=\frac{W_{E}I_{0}+w_{EE}\left\langle S^{\left( E \right)} \right\rangle+GJ\left\langle S^{\left( E \right)} \right\rangle-\left\langle I^{\left( E \right)} \right\rangle}{\left\langle S^{\left( I \right)} \right\rangle},\#\left( S17 \right) \end{aligned}$$

where the steady-state value $\left\langle S^{\left( I \right)} \right\rangle=H^{\left( I \right)}\left( \left\langle I^{\left( I \right)} \right\rangle\right)\tau_{I}$ was numerically solved using the expression

$$\begin{aligned} W_{I}I_{0}+w_{EI}\left\langle S^{\left( E \right)} \right\rangle-H^{\left( I \right)}\left( \left\langle I^{\left( I \right)} \right\rangle\right)\tau_{I}-\left\langle I^{\left( I \right)} \right\rangle=0.\#\left( S18 \right) \end{aligned}$$

Hence, the value of $w_{IE}$ dynamically changes as a function of the other free parameters: $w_{EE}$, $w_{EI}$, and $G$. Because finding the optimal parameter set on a multidimensional parameter space is computationally expensive, the analytic FC (see ‘Modelling resting-state dynamics’ in Methods) was used. Model fitting was done using the methods of Approximate Bayesian Computation and hierarchical Population Monte Carlo ^55,56^ that minimizes the distance between empirical and model FCs. See ^53^ for further details about the model optimization process.

S10. Lag threads algorithm

The lag threads algorithm starts by calculating the lagged cross-covariance function of the time series between brain regions. Assuming that BOLD-fMRI time series are aperiodic ^57^, the time lag (or delay) between regions where the cross-covariance function exhibits an extremum (typically between 0 to 2 s) was obtained ^58^. This resulted in an anti-symmetric time-delay matrix $TD$, with elements $\tau_{ij}$ corresponding to the time delay between regions $i$ and $j$, with column $i$ representing the lag map of the system with reference to region $i$, and $\tau_{ij}=-\tau_{ji}$. Therefore, $\tau_{ij}>0$ means that region $j$ lags behind region $i$. The underlying lag structure was quantified in two ways: (i) taking the mean time lag of each region (mean of each column of $TD$); and (ii) applying a PCA on $TD_{z}$, which is $TD$ with each column being zero-meaned. The first method obtains the average temporal ordering of brain regions, assuming that a single lag process governs the brain, which has been used in several past studies ^58–60^. However, for systems like the brain with multiple lag processes, the mean time lag cannot capture all fundamental lag patterns, which can be recovered via PCA ^61^. Here, we used the first two dominant PCs, explaining 74% of the variance of the empirical data. Thus, we calculated three lag projections: (i) mean lag; (ii) first PC (PC1 lag); and (iii) second PC (PC2 lag).

The $TD$ matrix was calculated on empirical and simulated resting-state BOLD-fMRI time courses parcellated using the HCP-MMP1 parcellation (see ‘Cortical parcellations’ in Methods). For the empirical data, a $TD$ matrix was calculated for each of the 255 HCP individuals, and then an average $TD$ matrix was calculated across individuals. For the simulated data, we generated time series for 255 trials (to match the 255 HCP individuals of the empirical data) of the wave and neural mass models using their respective original optimized parameters (see ‘Modelling resting-state dynamics in Methods’). A $TD$ matrix was calculated for each trial. Then, an average $TD$ matrix was calculated across trials. The average $TD$ matrices are shown in Extended Data Fig. 9a. Finally, the three lag projections were obtained from the empirical and simulated average $TD$ matrices (Extended Data Figs. 9b–d).

The results in Extended Data Fig. 9b show that the mean lag pattern of the wave model is significantly correlated with the empirical pattern, whereas the neural mass model’s mean lag pattern is not. For PC1 lag, the performance of the wave model slightly decreased but was still superior to the neural mass model (Extended Data Fig. 9c). Correlations between model and empirical patterns for PC2 lag (Extended Data Fig. 9d) were not significant, but the correlation was still higher for the wave model. These results show that, regardless of the method for calculating lag projections, the wave model captures the time-lagged properties of empirical fMRI data better than the neural mass model. This further highlights that wave dynamics can provide an accurate and physically mechanistic account of macroscale, resting-state dynamics, consistent with previous studies ^62–64^. We also emphasize that the performance of these models in capturing lag structure is likely to be a conservative estimate, as the models rely on a simple and spatially uniform hemodynamic forward model that does not account for regional variations in neurovascular coupling ^65–67^. Such variations are likely to strongly affect the empirically observed lags.

S11. Supplementary Discussion

The comparatively poor performance of connectome eigenmodes indicates that topologically complex long-range connections afford minimal benefit in accurately explaining cortical activity measured with fMRI. However, extensive evidence has shown that such connections may provide important functional and evolutionary advantages ^68–70^. Figure 2d of the main text alludes to these advantages, where low-order connectome eigenmodes perform slightly better in reconstructing task-activation maps at low frequencies. Low-order connectome eigenmodes comprise more complex spatial patterns than low-order geometric eigenmodes, which may afford greater flexibility in capturing spatially complex patterns of task activations. However, this advantage for connectome eigenmodes only persists for the first 25–30 modes, where reconstruction accuracies are generally low (i.e., *r* < 0.50) and do not differ substantially from the EDR eigenmodes.

Another potential aspect where long-range connections may come into play is the formation of cortical functional gradients. Our work shows that the geometric eigenmodes of subcortical structures have a near-perfect match to each structure’s FC-derived functional gradients. However, we do not observe the same one-to-one spatial correspondence between single geometric eigenmodes and previously described FC-derived functional gradients of the neocortex, the most dominant of which captures a hierarchical sensory-fugal axis of function ^71^. Functional gradients of the neocortex may thus reflect the complex contribution of long-range connections (e.g., subcortical-cortical connections) or a superposition of geometric modes ^37^, just as musical chords emerge from combinations of individual notes.

S12. Supplementary References

1. Beurle, R. L. Properties of a mass of cells capable of regenerating pulses. *Philosophical Transactions of the Royal Society of London. Series B, Biological Sciences* **240**, 55–94 (1956).

2. Lopes da Silva, F. H., van Rotterdam, A., Barts, P., van Heusden, E. & Burr, W. Models of Neuronal Populations: The Basic Mechanisms of Rhythmicity. *Progress in Brain Research* **45**, 281–308 (1976).

3. Wright, J. J. & Liley, D. T. J. Simulation of electrocortical waves. *Biological Cybernetics* **72**, 347–356 (1995).

4. Deco, G., Jirsa, V. K., Robinson, P. A., Breakspear, M. & Friston, K. The dynamic brain: From spiking neurons to neural masses and cortical fields. *PLoS Computational Biology* **4**, (2008).

5. Jirsa, V. & Haken, H. Field Theory of Electromagnetic Brain Activity. *Physical Review Letters* **77**, 960–963 (1996).

6. Robinson, P. A., Rennie, C. J. & Wright, J. J. Propagation and stability of waves of electrical activity in the cerebral cortex. *Physical Review E* **56**, 826–840 (1997).

7. Robinson, P. A., Rennie, C. J., Rowe, D. L., O’Connor, S. C. & Gordon, E. Multiscale brain modelling. *Philosophical Transactions of the Royal Society B: Biological Sciences* **360**, 1043–1050 (2005).

8. Sanz-Leon, P. *et al.* NFTsim: Theory and Simulation of Multiscale Neural Field Dynamics. *PLoS Computational Biology* **14**, e1006387 (2018).

9. Braitenberg, V. & Schüz, A. *Cortex: Statistics and Geometry of Neuronal Connectivity*. (Springer-Verlag Berlin, 1998).

10. Henderson, J. A. & Robinson, P. A. Relations between the geometry of cortical gyrification and white-matter network architecture. *Brain Connectivity* **4**, 112–130 (2014).

11. Robinson, P. A. Physical brain connectomics. *Physical Review E* **99**, 012421 (2019).

12. Robinson, P. A. Interrelating anatomical, effective, and functional brain connectivity using propagators and neural field theory. *Physical Review E* **85**, (2012).

13. Nunez, P. L. The brain wave equation: a model for the EEG. *Mathematical Biosciences* **21**, 279–297 (1974).

14. Robinson, P. A. *et al.* Prediction of electroencephalographic spectra from neurophysiology. *Physical Review E* **63**, 021903 (2001).

15. Pang, J. C. & Robinson, P. A. Neural mechanisms of the EEG alpha-BOLD anticorrelation. *NeuroImage* **181**, 461–470 (2018).

16. Rennie, C. J., Robinson, P. A. & Wright, J. J. Unified neurophysical model of EEG spectra and evoked potentials. *Biological Cybernetics* **86**, 457–471 (2002).

17. Mukta, K. N., Robinson, P. A., Pagès, J. C., Gabay, N. C. & Gao, X. Evoked response activity eigenmode analysis in a convoluted cortex via neural field theory. *Physical Review E* **102**, (2020).

18. Abeysuriya, R. G., Rennie, C. J. & Robinson, P. A. Physiologically based arousal state estimation and dynamics. *Journal of Neuroscience Methods* **253**, 55–69 (2015).

19. Assadzadeh, S. & Robinson, P. A. Necessity of the sleep-wake cycle for synaptic homeostasis: System-level analysis of plasticity in the corticothalamic system. *Royal Society Open Science* **5**, (2018).

20. Gabay, N. C., Babaie-Janvier, T. & Robinson, P. A. Dynamics of cortical activity eigenmodes including standing, traveling, and rotating waves. *Physical Review E* **98**, 042413 (2018).

21. Tokariev, A. *et al.* Large-scale brain modes reorganize between infant sleep states and carry prognostic information for preterms. *Nature Communications* **10**, 2619 (2019).

22. Nozari, E. *et al.* Is the brain macroscopically linear? A system identification of resting state dynamics. *arXiv* (2020).

23. Robinson, P. A. *et al.* Eigenmodes of brain activity: Neural field theory predictions and comparison with experiment. *NeuroImage* **142**, 79–98 (2016).

24. Robinson, P. A. *et al.* Determination of Dynamic Brain Connectivity via Spectral Analysis. *Frontiers in Human Neuroscience* **15**, (2021).

25. Gabay, N. C. & Robinson, P. A. Cortical geometry as a determinant of brain activity eigenmodes: Neural field analysis. *Physical Review E* **96**, (2017).

26. Nunez, P. L. *Neocortical Dynamics and Human EEG Rhythms*. (Oxford University Press, 1995).

27. Bressloff, P. C. Spatiotemporal dynamics of continuum neural fields. *Journal of Physics A: Mathematical and Theoretical* **45**, 033001 (2012).

28. Coombes, S., Beim Graben, P., Potthast, R. & Wright, J. J. *Neural fields: Theory and applications*. vol. 9783642545 (Springer, 2014).

29. Bick, C., Goodfellow, M., Laing, C. R. & Martens, E. A. Understanding the dynamics of biological and neural oscillator networks through exact mean-field reductions: a review. *Journal of Mathematical Neuroscience* **10**, (2020).

30. Barch, D. M. *et al.* Function in the human connectome: Task-fMRI and individual differences in behavior. *NeuroImage* **80**, 169–189 (2013).

31. Woolrich, M. W., Behrens, T. E. J., Beckmann, C. F., Jenkinson, M. & Smith, S. M. Multilevel linear modelling for FMRI group analysis using Bayesian inference. *NeuroImage* **21**, 1732–1747 (2004).

32. Robinson, E. C. *et al.* Multimodal surface matching with higher-order smoothness constraints. *NeuroImage* **167**, 453–465 (2018).

33. van Essen, D. C. *et al.* The WU-Minn Human Connectome Project: An overview. *NeuroImage* **80**, 62–79 (2013).

34. Smith, S. *et al.* The minimal preprocessing pipelines for the Human Connectome Project. *NeuroImage* **80**, 105–124 (2013).

35. Salimi-Khorshidi, G. *et al.* Automatic denoising of functional MRI data: combining independent component analysis and hierarchical fusion of classifiers. *Neuroimage* **15**, 449–468 (2013).

36. Mansour L, S., Tian, Y., Yeo, B. T. T., Cropley, V. & Zalesky, A. High-resolution connectomic fingerprints: Mapping neural identity and behavior. *NeuroImage* **229**, 117695 (2021).

37. Henderson, J. A., Aquino, K. M. & Robinson, P. A. Empirical estimation of the eigenmodes of macroscale cortical dynamics: Reconciling neural field eigenmodes and resting-state networks. *Neuroimage: Reports* **2**, 100103 (2022).

38. Chen, Y.-C. *et al.* The individuality of shape asymmetries of the human cerebral cortex. *eLife* **11**, e75056 (2022).

39. Atasoy, S., Donnelly, I. & Pearson, J. Human brain networks function in connectome-specific harmonic waves. *Nature Communications* **7**, 10340 (2016).

40. Naze, S., Proix, T., Atasoy, S. & Kozloski, J. R. Robustness of connectome harmonics to local gray matter and long-range white matter connectivity changes: Sensitivity analysis of Connectome Harmonics. *NeuroImage* **224**, 117364 (2021).

41. Zalesky, A. *et al.* Whole-brain anatomical networks: Does the choice of nodes matter? *NeuroImage* **50**, 970–983 (2010).

42. Fornito, A., Zalesky, A. & Bullmore, E. T. *Fundamentals of Brain Network Analysis*. (2016).

43. Smith, S. M., Hyvärinen, A., Varoquaux, G., Miller, K. L. & Beckmann, C. F. Group-PCA for very large fMRI datasets. *NeuroImage* **101**, 738–749 (2014).

44. Do Carmo, M. P. *Differential Geometry of Curves and Surfaces: Revised and Updated Second Edition*. (Dover Publications, 2016).

45. Klingenberg, W. P. A. *Riemannian Geometry*. (de Gruyter, 1995).

46. Reuter, M. *Laplace Spectra for Shape Recognition*. (Books On Demand, 2006).

47. Strichartz, R. S. Harmonic analysis as spectral theory of Laplacians. *Journal of Functional Analysis* **87**, 51–148 (1989).

48. Chung, F. R. K. *Spectral Graph Theory*. (American Mathematical Society, 1996).

49. Carp, J. The secret lives of experiments: Methods reporting in the fMRI literature. *NeuroImage* **63**, 289–300 (2012).

50. Coalson, T. S., Van Essen, D. C. & Glasser, M. F. The impact of traditional neuroimaging methods on the spatial localization of cortical areas. *Proceedings of the National Academy of Sciences of the United States of America* **115**, E6356–E6365 (2018).

51. Robinson, P. A. Interrelating anatomical, effective, and functional brain connectivity using propagators and neural field theory. *Physical Review E* **85**, (2012).

52. Arfken, G. *Mathematical Methods for Physicists*. (Academic Press Inc, 1985).

53. Demirtaş, M. *et al.* Hierarchical Heterogeneity across Human Cortex Shapes Large-Scale Neural Dynamics. *Neuron* **101**, 1181–1194 (2019).

54. Deco, G. *et al.* How local excitation-inhibition ratio impacts the whole brain dynamics. *Journal of Neuroscience* **34**, 7886–7898 (2014).

55. Beaumont, M. A., Cornuet, J. M., Marin, J. M. & Robert, C. P. Adaptive approximate Bayesian computation. *Biometrika* **96**, 983–990 (2009).

56. Turner, B. M. & Van Zandt, T. Hierarchical Approximate Bayesian Computation. *Psychometrika* **79**, 185–209 (2014).

57. He, B. J., Zempel, J. M., Snyder, A. Z. & Raichle, M. E. The Temporal Structures and Functional Significance of Scale-free Brain Activity. *Neuron* **66**, 353–369 (2010).

58. Mitra, A., Snyder, A. Z., Hacker, C. D. & Raichle, M. E. Lag structure in resting-state fMRI. *Journal of Neurophysiology* **111**, 2374–2391 (2014).

59. Raut, R. V. *et al.* Global waves synchronize the brain’s functional systems with fluctuating arousal. *Science Advances* **7**, (2021).

60. Bolt, T. *et al.* A parsimonious description of global functional brain organization in three spatiotemporal patterns. *Nature Neuroscience* **25**, 1093–1103 (2022).

61. Mitra, A., Snyder, A. Z., Blazey, T. & Raichle, M. E. Lag threads organize the brain’s intrinsic activity. *Proceedings of the National Academy of Sciences* **112**, E2235–E2244 (2015).

62. Majeed, W. *et al.* Spatiotemporal dynamics of low frequency BOLD fluctuations in rats and humans. *NeuroImage* **54**, 1140–1150 (2011).

63. Matsui, T., Murakami, T. & Ohki, K. Transient neuronal coactivations embedded in globally propagating waves underlie resting-state functional connectivity. *Proceedings of the National Academy of Sciences* **113**, 6556–6561 (2016).

64. Chan, A. W., Mohajerani, M. H., LeDue, J. M., Wang, Y. T. & Murphy, T. H. Mesoscale infraslow spontaneous membrane potential fluctuations recapitulate high-frequency activity cortical motifs. *Nature Communications* **6**, 7738 (2015).

65. Aquino, K. M., Schira, M. M., Robinson, P. A., Drysdale, P. M. & Breakspear, M. Hemodynamic traveling waves in human visual cortex. *PLoS Computational Biology* **8**, (2012).

66. Pang, J. C., Robinson, P. A., Aquino, K. M. & Vasan, N. Effects of astrocytic dynamics on spatiotemporal hemodynamics: Modeling and enhanced data analysis. *NeuroImage* **147**, 994–1005 (2017).

67. Pang, J. C., Aquino, K. M., Robinson, P. A., Lacy, T. C. & Schira, M. M. Biophysically based method to deconvolve spatiotemporal neurovascular signals from fMRI data. *Journal of Neuroscience Methods* **308**, 6–20 (2018).

68. van den Heuvel, M. P., Kahn, R. S., Goñi, J. & Sporns, O. High-cost, high-capacity backbone for global brain communication. *Proceedings of the National Academy of Sciences of the United States of America* **109**, 11372–11377 (2012).

69. Arnatkeviciute, A. *et al.* Genetic influences on hub connectivity of the human connectome. *Nature Communications* **12**, (2021).

70. Pang, J. C., Rilling, J. K., Roberts, J. A., van den Heuvel, M. P. & Cocchi, L. Evolutionary shaping of human brain dynamics. *eLife* **11**, e80627 (2022).

71. Margulies, D. S. *et al.* Situating the default-mode network along a principal gradient of macroscale cortical organization. *Proceedings of the National Academy of Sciences of the United States of America* **113**, 12574–12579 (2016).

S13. Supplementary Figures

**
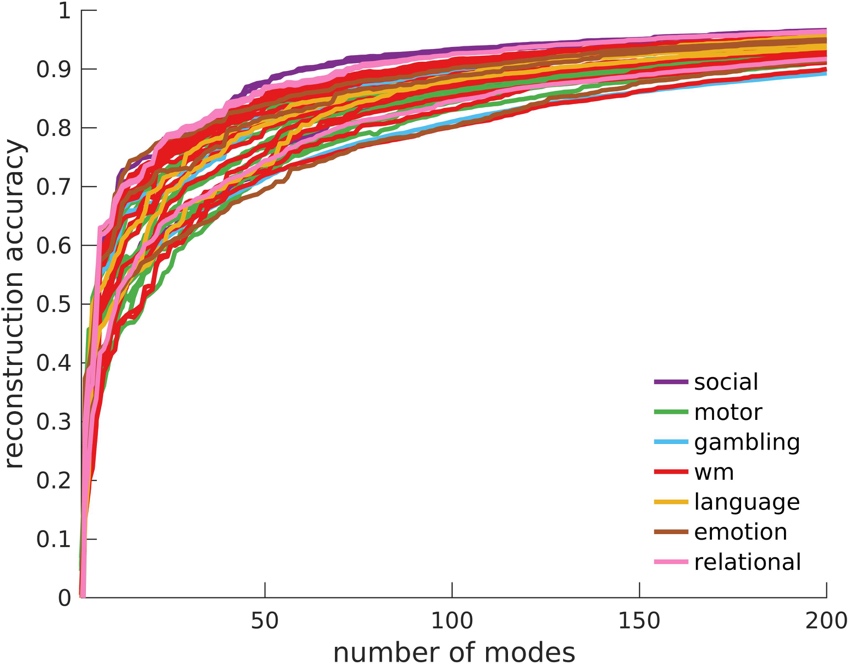
**

**Supplementary Fig. 1. Reconstruction accuracy of 47 HCP task-contrast maps obtained with geometric eigenmodes.** The lines are coloured according to the groups defined by the 7 broad HCP task types (Section S2.1 and Supplementary Table 2). wm = working memory.

**
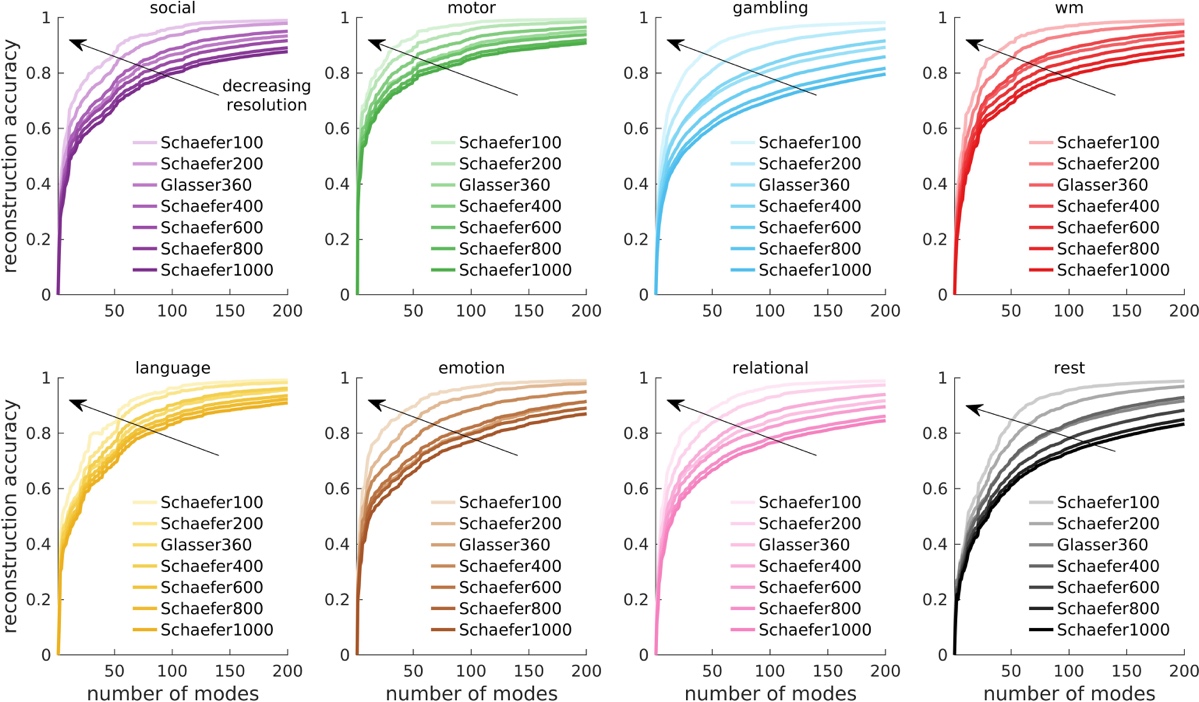
**

**Supplementary Fig. 2. Reconstruction accuracy of 7 key HCP task-contrast maps and resting-state FC for different parcellation resolutions.** See Section S2.1 and Supplementary Table 2 for details about the contrast maps. wm = working memory. The lines with dark to light colours represent decreasing parcellation resolutions (direction of arrows); i.e., Schaefer100, Schaefer200, Glasser360, Schaefer400, Schaefer600, Schaefer800, and Schaefer1000 has 100, 200, 360, 400, 600, 800, and 1000 parcels, respectively, across both hemispheres.

**
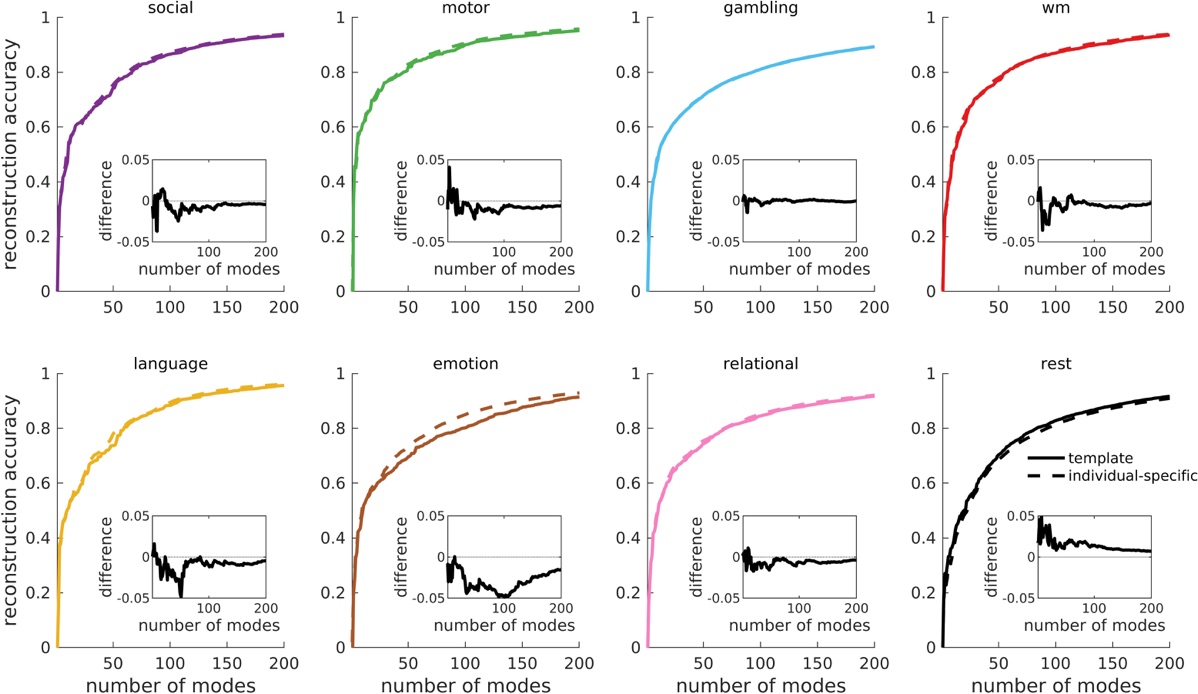
**

**Supplementary Fig. 3. Reconstruction accuracy of 7 key HCP task-contrast maps and resting-state FC using template-derived and individual-specific geometric eigenmodes.** See Section S2.1 and Supplementary Table 2 for details about the contrast maps. wm = working memory. The solid lines represent results achieved by eigenmodes derived from a template surface (Fig. 1d). The dashed lines represent results achieved by individual-specific eigenmodes derived from individual surfaces. The insets show the difference between the two results (i.e., template minus individual-specific).

**
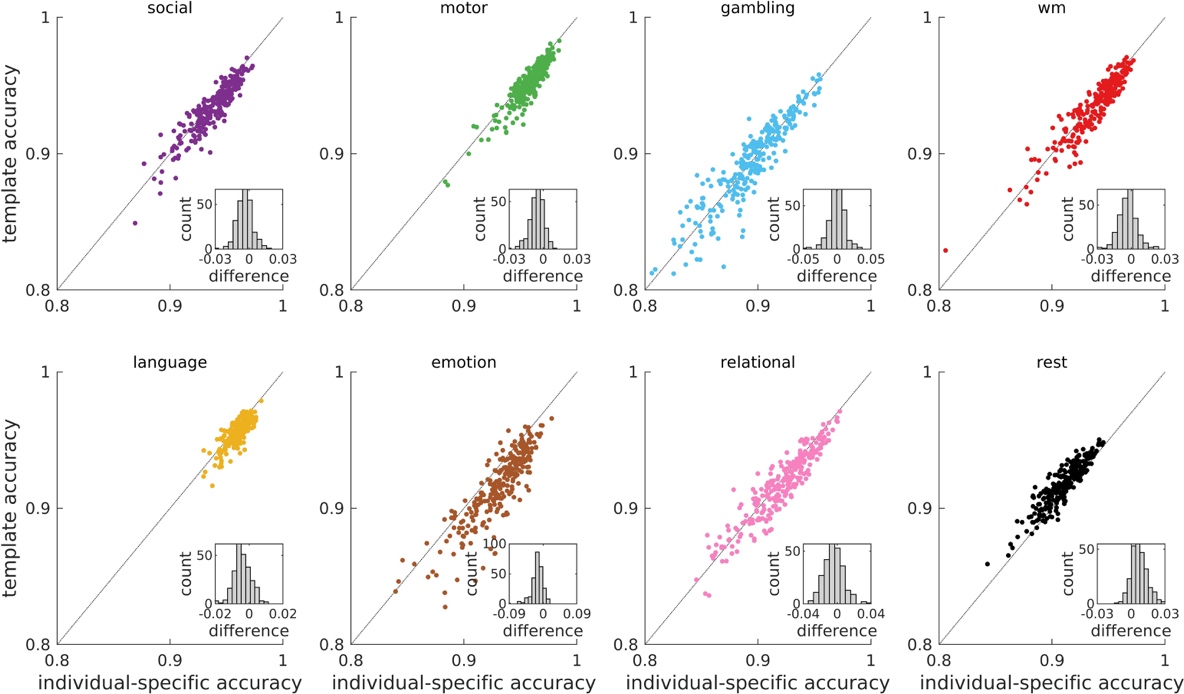
**

**Supplementary Fig. 4. Individual-based reconstruction accuracy of 7 key HCP task-contrast maps and resting-state FC using 200 template-derived and individual-specific geometric eigenmodes.** See Section S2.1 and Supplementary Table 2 for details about the contrast maps. wm = working memory. Template eigenmodes were derived from a template surface, while individual-specific eigenmodes were derived from individual surfaces. Each point corresponds to an individual. The dotted lines represent the template accuracy = individual-specific accuracy lines. Points above the dotted lines mean that template accuracy > individual-specific accuracy. The insets show the histogram of the difference between the reconstruction accuracy achieved by template-derived and individual-specific eigenmodes (i.e., template minus individual-specific).

**
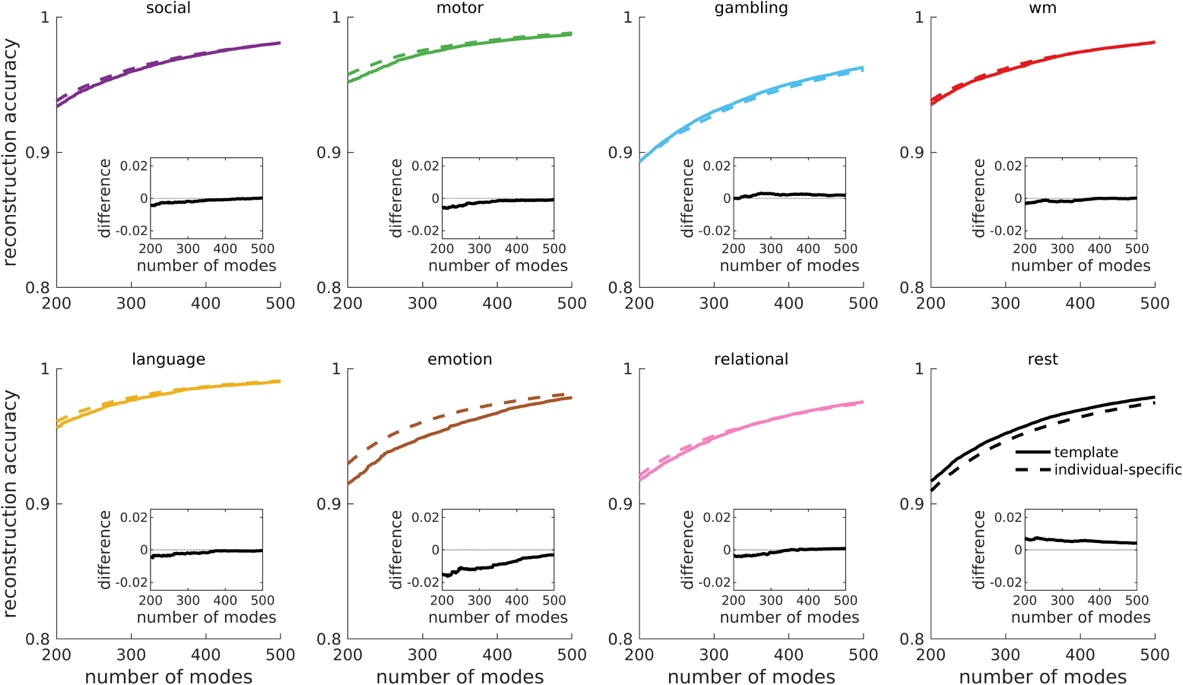
**

**Supplementary Fig. 5. Reconstruction accuracy of 7 key HCP task-contrast maps and resting-state FC using 200 to 500 template-derived and individual-specific geometric eigenmodes.** See Section S2.1 and Supplementary Table 2 for details about the contrast maps. wm = working memory. The solid lines represent results achieved by eigenmodes derived from a template surface (Fig. 1d). The dashed lines represent results achieved by individual-specific eigenmodes derived from individual surfaces. The insets show the difference between the reconstruction accuracy achieved by template-derived and individual-specific eigenmodes (i.e., template minus individual-specific).

**
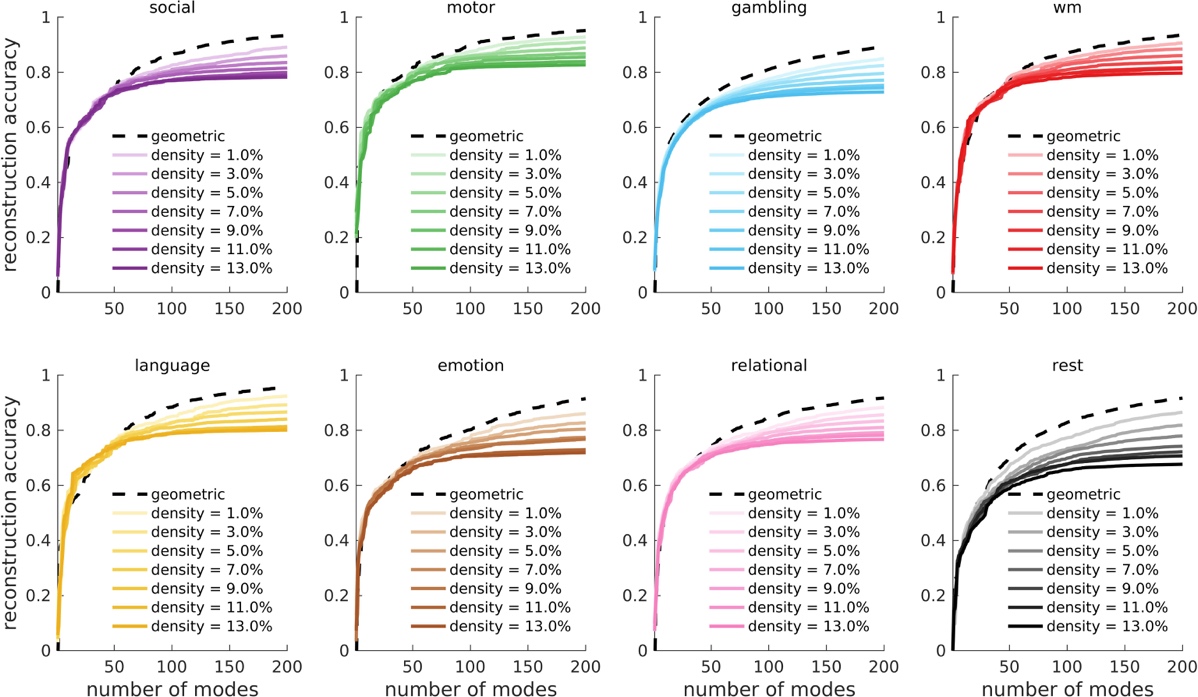
**

**Supplementary Fig. 6. Reconstruction accuracy of 7 key HCP task-contrast maps and resting-state FC achieved by geometric eigenmodes and connectome eigenmodes of varying connectome densities.** See Section S2.1 and Supplementary Table 2 for details about the contrast maps. wm = working memory. The dashed lines represent results achieved by geometric eigenmodes (Fig. 1d). The lines with light to dark colours represent results achieved by connectome eigenmodes using a connectivity matrix of increasing densities (from 1.0% to 13.0%).

**
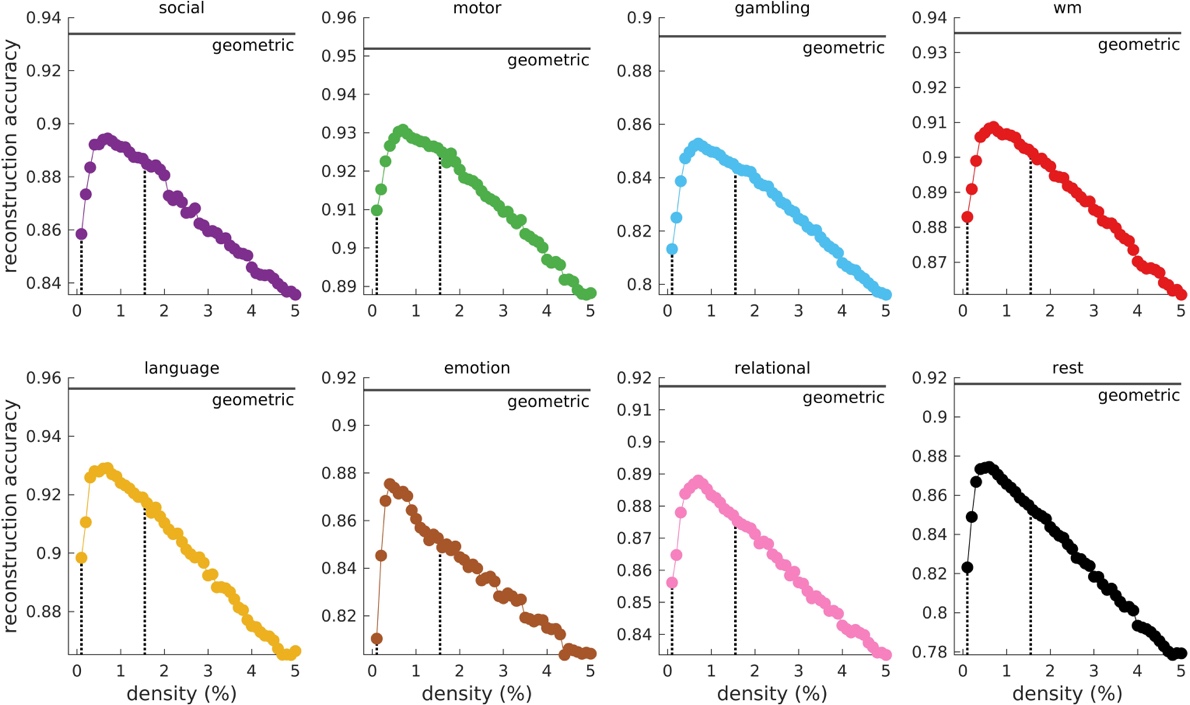
**

**Supplementary Fig. 7. Reconstruction accuracy of 7 key HCP task-contrast maps and resting-state FC using 200 connectome eigenmodes of varying connectome densities.** See Section S2.1 and Supplementary Table 2 for details about the contrast maps. wm = working memory. The solid line corresponds to the reconstruction accuracy achieved by 200 geometric eigenmodes. The dotted lines correspond to connectome densities of 0.1% and 1.55% used to generate the connectome and density-matched connectome eigenmodes in Extended Data Fig. 1, respectively.

**
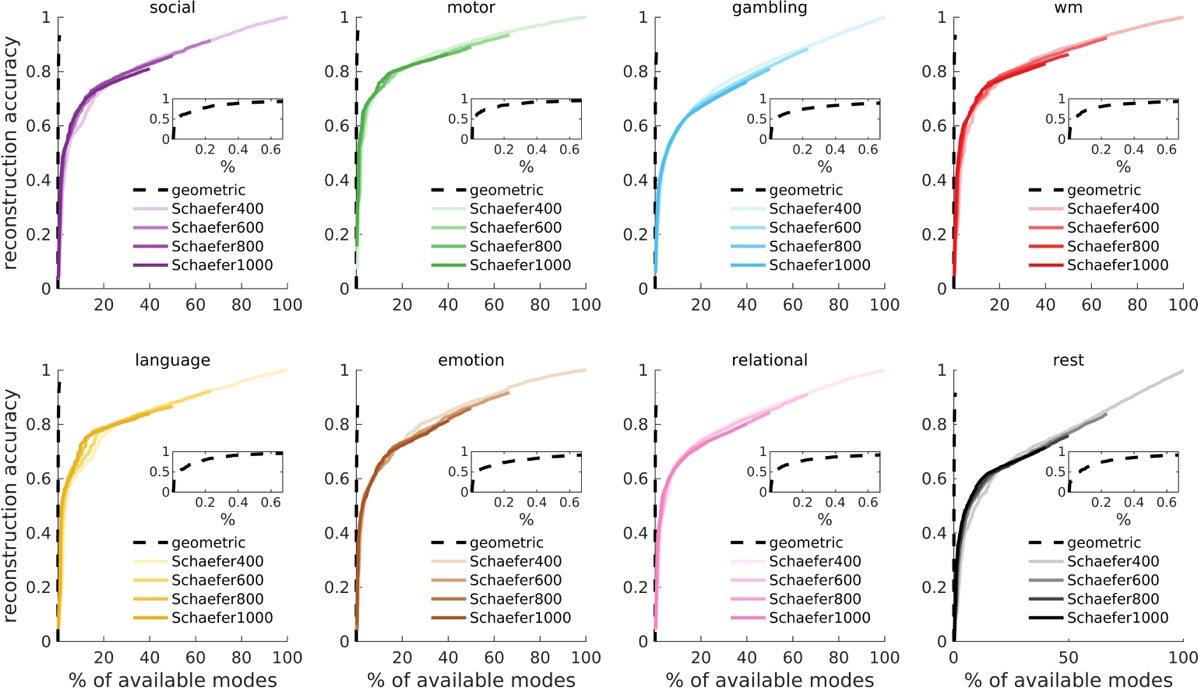
**

**Supplementary Fig. 8. Reconstruction accuracy of 7 key HCP task-contrast maps and resting-state FC achieved by geometric eigenmodes and discrete connectome eigenmodes for different parcellation resolutions.** See Section S2.1 and Supplementary Table 2 for details about the contrast maps. wm = working memory. For all cases, we use a maximum of 200 modes to directly compare the results to the rest of the study. The dashed lines represent results achieved by geometric eigenmodes (Fig. 1d); see magnified version in the insets. The lines with light to dark colours represent results achieved by discrete connectome eigenmodes using a connectivity matrix parcellated at increasing resolutions. The reconstruction accuracies are plotted versus the percentage of modes used with respect to the dimension of each full basis set (i.e., total number of available modes), which corresponds to the number of vertices of the cortical surface (for geometric eigenmodes) or number of parcels in each hemisphere (for discrete connectome eigenmodes). Hence, for the geometric eigenmodes, we use the first 200 modes out of 32,492 available modes per hemisphere, which is why the dotted lines terminate at 0.6% of available modes. For the discrete connectome eigenmodes using parcellations of Schaefer400, Schaefer600, Schaefer800, and Schaefer1000, we use the first 200 modes out of 200, 300, 400, and 500 available modes per hemisphere, respectively. Hence, the solid lines terminate at 100%, 66.7%, 0.50%, and 0.40% of available modes, respectively. The results emphasize that geometric eigenmodes provide the most parsimonious and compact representation of brain activity.

**
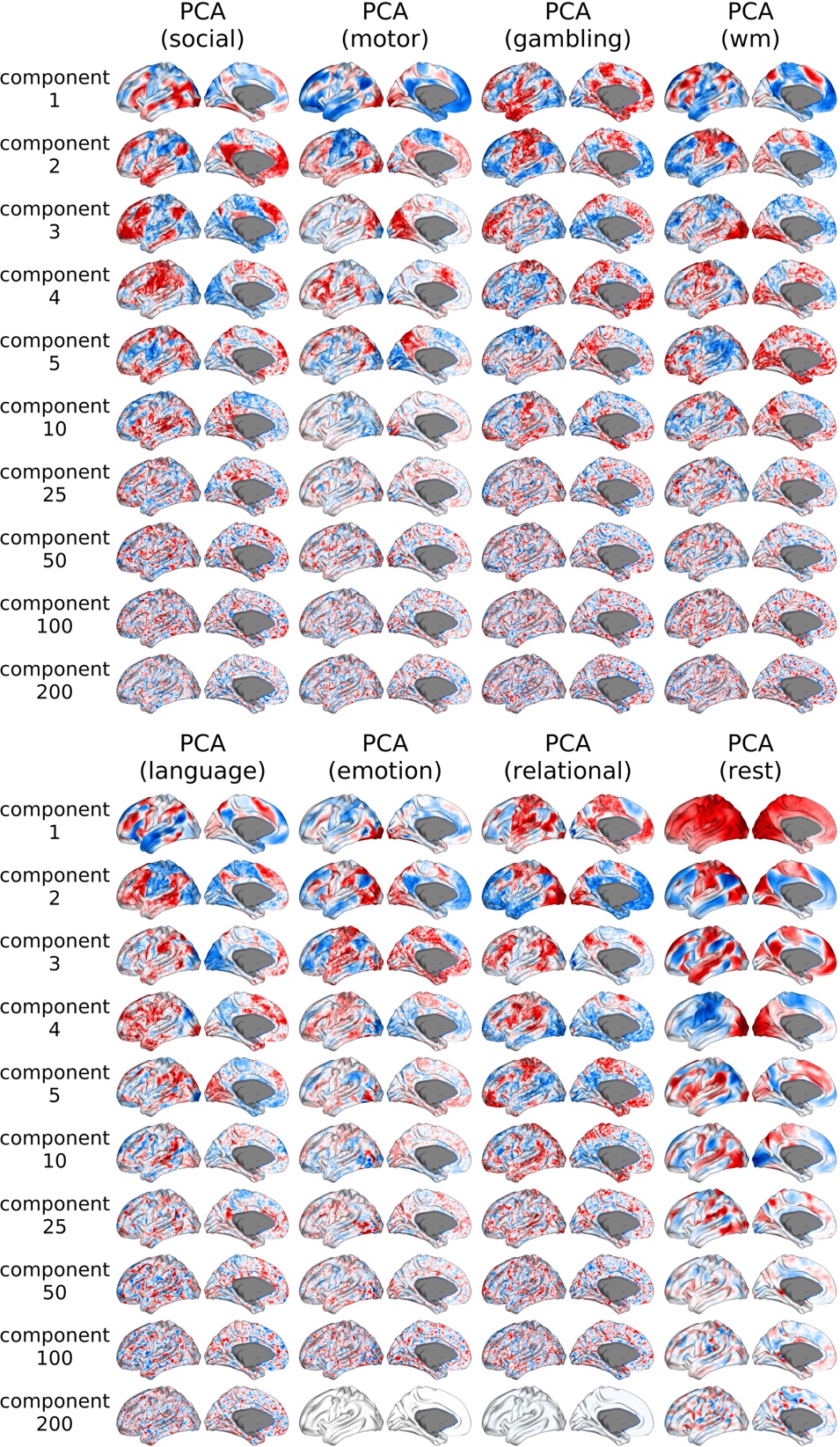
**

**Supplementary Fig. 9. Principal components obtained via principal component analysis (PCA) of fMRI data.** The PCAs are trained on each of the 7 key HCP task-contrast maps and resting-state time series of 200 individuals. See Section 2.1 and Supplementary Table 2 for details about the contrast maps. wm = working memory. Principal components 1–5, 10, 25, 50, 100, and 200 are shown from top to bottom. Negative–zero–positive values are coloured as blue–white–red.


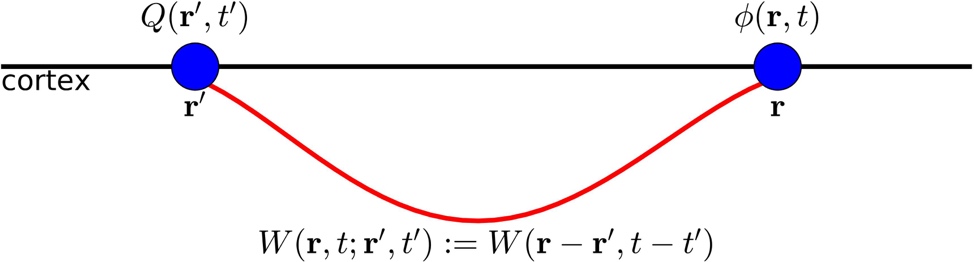


**Supplementary Fig. 10. Schematic of signal propagation in neural field theory.** Two points on the cortex at locations $\boldsymbol{r'}$ and $\boldsymbol{r}$ are connected by a white-matter tract (red curve). For an isotropic medium, the activity $\phi\left( \boldsymbol{r},t \right)$ is a convolution of the source $Q\left( \boldsymbol{r'},t' \right)$ and a connectivity kernel $W\left( \boldsymbol{r},t;\boldsymbol{r'},t' \right)$ imposed by the white-matter connection, which depends only on the spatial separation, $\boldsymbol{r}-\boldsymbol{r'}$, and the time separation, $t-t'$.

**
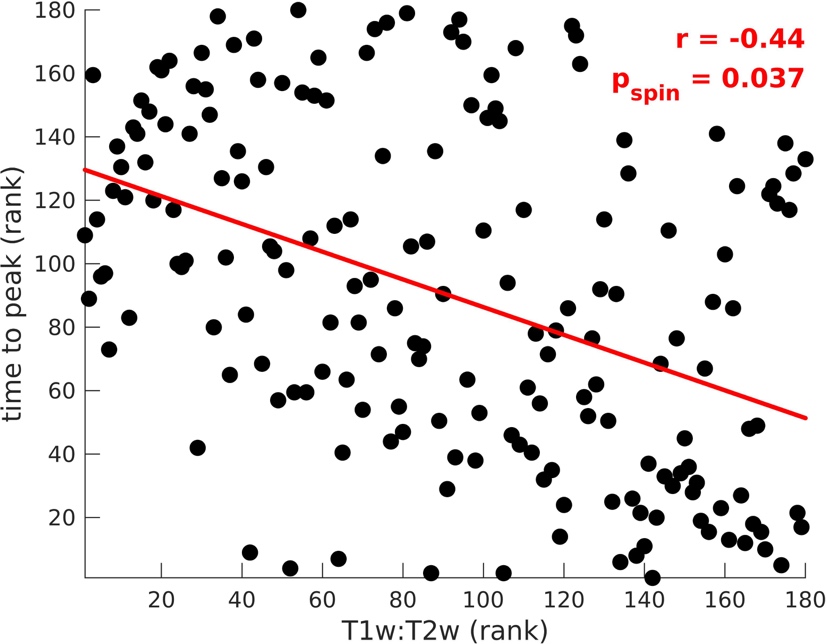
**

**Supplementary Fig. 11. Comparison of time to peak activation and T1w:T2w of each region.** Relationship of the ranked activity profile time to peak and ranked T1w:T2w value of all 180 brain regions in one hemisphere. The red line represents a linear fit of the ranked variables with Spearman correlation coefficient *r* and one-sided spin-test *p*-value, *p*_spin_, estimated from 10,000 permutations.

S14. Supplementary Tables

**Supplementary Table 1. Spatial wavelengths of the eigenmodes.**

| **Eigengroup** | **Wavelength (mm)** | **Eigenmodes included in the eigengroup** |
| --- | --- | --- |
| 0 | $-$ | 1 |
| 1 | 297.7 | 2–4 |
| 2 | 171.9 | 5–9 |
| 3 | 121.5 | 10–16 |
| 4 | 94.1 | 17–25 |
| 5 | 76.9 | 26–36 |
| 6 | 65.0 | 37–49 |
| 7 | 56.3 | 50–64 |
| 8 | 49.6 | 65–81 |
| 9 | 44.4 | 82–100 |
| 10 | 40.1 | 101–121 |
| 11 | 36.6 | 122–144 |
| 12 | 33.7 | 145–169 |
| 13 | 31.2 | 170–196 |
| 14 | 29.1 | 197–225 |

**Supplementary Table 2. HCP task contrasts.**

| **Task type** | **Number of contrasts** | **Contrasts** | **Key contrast** |
| --- | --- | --- | --- |
| social | 3 | random; tom; tom_random | tom_random |
| motor | 13 | cue; lf; lh; rf; rh; t; avg; lf_avg; lh_avg; rf_avg; rh_avg; t_avg; cue_avg | cue_avg |
| gambling | 3 | punish; reward; punish_reward | punish_reward |
| working memory (wm) | 19 | 2bk_body; 2bk_face; 2bk_place; 2bk_tool; 0bk_body; 0bk_face; 0bk_place; 0bk_tool; 2bk; 0bk; body; face; place; tool; body_avg; face_avg; place_avg; tool_avg; 2bk_0bk | 2bk_0bk |
| language | 3 | math; story; math_story | math_story |
| emotion | 3 | faces; shapes; faces_shapes | faces_shapes |
| relational | 3 | match; rel; match_rel | match_rel |

**Supplementary Table 3. Fixed and free parameters of the BEI neural mass model.**

| **Fixed parameters** | | **Free parameters** |
| --- | --- | --- |
| **Symbol** | **Value** | **Symbol** |
| $\tau_{E}$ | 0.1 s | $w_{EE}$ |
| $\tau_{I}$ | 0.01 s | $w_{IE}$ |
| $\gamma$ | 0.641 | $w_{EI}$ |
| $\sigma$ | 0.01 nA | $G$ |
| $a_{E}$ | 310 nC^-1^ |  |
| $b_{E}$ | 125 s^-1^ |  |
| $d_{E}$ | 0.16 s |  |
| $a_{I}$ | 615 nC^-1^ |  |
| $b_{I}$ | 177 s^-1^ |  |
| $d_{I}$ | 0.087 s |  |
| $I^{ext}$ | 0 nA |  |
| $I_{0}$ | 0.382 nA |  |
| $W_{E}$ | 1.0 |  |
| $W_{I}$ | 0.7 |  |
| $J$ | 0.15 nA |  |

S15. Supplementary Video

**Supplementary Video 1. Stimulus-evoked activity.** Wave propagation of activity after a 1 ms stimulation of the primary visual cortex (V1) from *t* = 1 to 2 ms.
