# Supplementary material for: Geometric constraints on human brain function
Source: Nature. 2023 May 31;618(7965):566–74. doi: 10.1038/s41586-023-06098-1 (PMC10266981; doi:10.1038/s41586-023-06098-1)
Supplement: Supplementary file 2 — Reporting Summary [file 41586_2023_6098_MOESM2_ESM.pdf]

nature portfolio

Corresponding author(s): James C. Pang
Last updated by author(s): Apr 5, 2023

Reporting Summary

Nature Portfolio wishes to improve the reproducibility of the work that we publish. This form provides structure for consistency and transparency in reporting. For further information on Nature Portfolio policies, see our Editorial Policies and the Editorial Policy Checklist.

Statistics

For all statistical analyses, confirm that the following items are present in the figure legend, table legend, main text, or Methods section.

n/a	Confirmed	
☐	☒	The exact sample size (*n*) for each experimental group/condition, given as a discrete number and unit of measurement
☐	☒	A statement on whether measurements were taken from distinct samples or whether the same sample was measured repeatedly
☐	☒	The statistical test(s) used AND whether they are one- or two-sided<br>*Only common tests should be described solely by name; describe more complex techniques in the Methods section.*
☐	☒	A description of all covariates tested
☐	☒	A description of any assumptions or corrections, such as tests of normality and adjustment for multiple comparisons
☐	☒	A full description of the statistical parameters including central tendency (e.g. means) or other basic estimates (e.g. regression coefficient) AND variation (e.g. standard deviation) or associated estimates of uncertainty (e.g. confidence intervals)
☐	☒	For null hypothesis testing, the test statistic (e.g. *F*, *t*, *r*) with confidence intervals, effect sizes, degrees of freedom and *P* value noted<br>*Give P values as exact values whenever suitable.*
☒	☐	For Bayesian analysis, information on the choice of priors and Markov chain Monte Carlo settings
☒	☐	For hierarchical and complex designs, identification of the appropriate level for tests and full reporting of outcomes
☐	☒	Estimates of effect sizes (e.g. Cohen's *d*, Pearson's *r*), indicating how they were calculated

Our web collection on statistics for biologists contains articles on many of the points above.

Software and code

Policy information about availability of computer code

Data collection

Analyses were undertaken on human neuroimaging data from the open-access dataset of the Human Connectome Project (HCP) at https://db.humanconnectome.org/. See Van Essen et al. (2013, NeuroImage) and Barch et al. (2013, NeuroImage) for further details.

Data analysis

Custom-written computer codes (MATLAB R2019b; Python 3.7) to calculate the eigenmodes, analyse results, perform other computational analyses, and reproduce the figures of the study are openly available at the GitHub repository https://github.com/NSBLab/BrainEigenmodes.

Additionally, the following open-source software packages were used in the study:
- FreeSurfer (v7.1.1)
- FSL (v6.0.4)
- gmsh (v3.0.3)
- connectome workbench (v1.5.0)
- HCP minimal preprocessing pipeline (Glasser et al., 2013, NeuroImage)
- HCP diffusion preprocessing pipeline (v3.19.0)
- Python libraries Nilearn, LaPy

For manuscripts utilizing custom algorithms or software that are central to the research but not yet described in published literature, software must be made available to editors and reviewers. We strongly encourage code deposition in a community repository (e.g. GitHub). See the Nature Portfolio guidelines for submitting code & software for further information.

Data

Policy information about availability of data

All manuscripts must include a data availability statement. This statement should provide the following information, where applicable:

- Accession codes, unique identifiers, or web links for publicly available datasets
- A description of any restrictions on data availability
- For clinical datasets or third party data, please ensure that the statement adheres to our policy

Raw and preprocessed MRI data were taken from the open-access Human Connectome Project (HCP) dataset at https://db.humanconnectome.org/. See Van Essen et al. (2013, NeuroImage) and Barch et al. (2013, NeuroImage) for further details. The dataset was acquired by the WU-Minn HCP consortium, with access provided to anyone agreeing to their data use terms.

10,000 human task-activation maps were taken from the open-access repository of NeuroVault (http://neurovault.org/).

All source data to generate the results of the study are openly available at Github repository https://github.com/NSBLab/BrainEigenmodes and the open science framework repository https://osf.io/xczmp/.

Field-specific reporting

Please select the one below that is the best fit for your research. If you are not sure, read the appropriate sections before making your selection.

☒ Life sciences ☐ Behavioural & social sciences ☐ Ecological, evolutionary & environmental sciences

For a reference copy of the document with all sections, see nature.com/documents/nr-reporting-summary-flat.pdf

Life sciences study design

All studies must disclose on these points even when the disclosure is negative.

Sample size	No new data were collected. We used data from 255 participants from the HCP dataset, which is the largest available unrelated sample with complete functional MRI data.
Data exclusions	Out of 1113 available human participants from the HCP dataset, we excluded participants that are related either as twins or siblings and do not have a complete set of task-evoked and resting-state functional MRI data.
Replication	N/A
Randomization	Participants were not allocated to experimental groups. Hence, randomization into groups was not applicable.
Blinding	Participants were not allocated to experimental groups and the study did not involve hypothesis testing. Hence, blinding was not applicable.

Reporting for specific materials, systems and methods

We require information from authors about some types of materials, experimental systems and methods used in many studies. Here, indicate whether each material, system or method listed is relevant to your study. If you are not sure if a list item applies to your research, read the appropriate section before selecting a response.

Materials & experimental systems

n/a	Involved in the study	
☒	☐	Antibodies
☒	☐	Eukaryotic cell lines
☒	☐	Palaeontology and archaeology
☒	☐	Animals and other organisms
☐	☒	Human research participants
☒	☐	Clinical data
☒	☐	Dual use research of concern

Methods

n/a	Involved in the study	
☒	☐	ChIP-seq
☒	☐	Flow cytometry
☐	☒	MRI-based neuroimaging

Human research participants

Policy information about studies involving human research participants

Population characteristics	All data were taken from the open-access HCP dataset. The participants comprised 255 healthy young adults (ages 22-35; 132 females).

Recruitment	See van Essen et al. (2013, NeuroImage) for recruitment details.
Ethics oversight	The open-access HCP dataset were acquired by the WU-Minn HCP consortium with local oversighting ethics committee approval and shared with the authors according to HCP's data use terms. All our procedures were carried out in accordance with protocols set by these data use terms. See van Essen et al. (2013; NeuroImage) for details of oversighting ethics committee.

Note that full information on the approval of the study protocol must also be provided in the manuscript.

Magnetic resonance imaging

Experimental design

Design type	No new data were collected. See van Essen et al. (2013, NeuroImage) and Barch et al. (2013, NeuroImage) for details.
Design specifications	No new data were collected. See van Essen et al. (2013, NeuroImage) and Barch et al. (2013, NeuroImage) for details.
Behavioral performance measures	No new data were collected. See van Essen et al. (2013, NeuroImage) and Barch et al. (2013, NeuroImage) for details.

Acquisition

Imaging type(s)	functional MRI and diffusion MRI
Field strength	3T
Sequence & imaging parameters	For functional MRI data (van Essen et al., 2013, NeuroImage; Barch et al., 2013, NeuroImage): gradient-echo EPI, FOV of 208x180 mm^2, matrix size of 104x90, isotropic voxel size of 2 mm, TR of 1720 ms, TE of 33.1 ms, flip angle of 52 deg.<br><br>For diffusion MRI data (van Essen et al., 2013, NeuroImage; Barch et al., 2013, NeuroImage): spin-echo EPI, FOV of 210x210 mm^2, matrix size of 140x140, isotropic voxel size of 1.25 mm, TR of 5520 ms, TE of 89.5 ms.
Area of acquisition	Whole-brain scan

Diffusion MRI ☒ Used ☐ Not used

Parameters	90 diffusion directions, b-weightings of 1000, 2000, 3000 s/mm^2, 174 slices

Preprocessing

Preprocessing software	Already preprocessed MRI data were used based on the HCP minimal preprocessing pipeline and HCP diffusion preprocessing pipeline (Glasser et al., 2013, NeuroImage). Preprocessing involved a combination of tools from FSL, FreeSurfer, and Connectome Workbench.
Normalization	Already normalized MRI data were used based on the HCP minimal preprocessing pipeline (Glasser et al., 2013, NeuroImage). Normalization included spatially alignment to the MNI standard space using FSL FNIRT and then data were projected onto the cortical surface.
Normalization template	All data were projected onto the fsLR cortical template surface with 32,492 vertices per hemisphere.
Noise and artifact removal	Already preprocessed MRI data were used based on the HCP minimal preprocessing pipeline and HCP diffusion preprocessing pipeline (Glasser et al., 2013, NeuroImage). Preprocessing of resting-state fMRI data included ICA-FIX.
Volume censoring	No volume censoring was performed.

Statistical modeling & inference

Model type and settings	Pearson correlation was used in Figs. 4b, 5a, 5b, 5c, 5d, 5e, 5f and Extended Data Figs. 9b, 9c, 9d.<br><br>Spearman rank correlation was used in Fig. 4e and Supplementary Fig. 11.<br><br>Kolmogorov-Smirnov test was used in Fig. 4b.
Effect(s) tested	For Pearson correlation:<br>Association between edge and node FC properties of model (simulated) and empirical data (Fig. 4b).<br>Association between the first three functional gradients and geometric eigenmodes (Figs. 5a, 5b, 5c).<br>Association between all pairs of functional gradients and geometric eigenmodes (Figs. 5d, 5e, 5f).<br>Association between lag properties of model (simulated) and empirical resting-state time series data (Extended Data Figs. 9b, 9c, 9d).<br><br>For Spearman rank correlation:<br>Association between T1w:T2w and response time to peak across regions of interest (Fig. 4e).<br>Association between T1w:T2w and response time to peak across the whole brain (Supplementary Fig. 11).

For Kolmogorov-Smirnov test:
Comparison of the distribution of FCD statistics of model (simulated) and empirical data (Fig. 4b).

Specify type of analysis: ☐ Whole brain ☐ ROI-based ☒ Both

Anatomical location(s)	The following atlas-based parcellations were used:<br>HCP-MMP1 atlas (Glasser et al., 2016, Nature) and Schaefer atlas (Schaefer et al., 2018, Cerebral Cortex).
Statistic type for inference<br>(See Eklund et al. 2016)	No inferences were made.
Correction	No corrections were performed.

Models & analysis

n/a	Involved in the study
☐	☒ Functional and/or effective connectivity
☒	☐ Graph analysis
☒	☐ Multivariate modeling or predictive analysis

Functional and/or effective connectivity	Pearson correlation.
